# The economics of sex work and major sporting events: Learning from the 2010 FIFA World Cup in South Africa

**DOI:** 10.1016/j.ssaho.2022.100251

**Published:** 2022

**Authors:** Jacob Kazungu, Marlise Richter, Stanley Luchters, Matthew F. Chersich, Matthew Quaife

**Affiliations:** a Health Economics Research Unit, KEMRI | Wellcome Trust Research Programme, Nairobi, Kenya; b Department of Global Health and Development, London School of Hygiene and Tropical Medicine, UK; c African Centre for Migration & Society, University of the Witwatersrand, Johannesburg, South Africa; d Alan J Flisher Centre for Public Mental Health, University of Cape Town, Cape Town, South Africa; e Department of Population Health, Aga Khan University, Nairobi, Kenya; f Burnet Institute, Melbourne, Australia; g Department of Epidemiology and Preventive Medicine, Monash University, Melbourne, Australia; h Department of Public Health and Primary Care, Ghent University, Ghent, Belgium; i Wits Reproductive Health and HIV Institute, Faculty of Health Science, University of the Witwatersrand, South Africa

**Keywords:** World Cup, South Africa, Economics of sex work, Price premiums, Price of sex, Condomless sex

## Abstract

Risk-taking in sex work is related to financial gains from condom-protected and condomless-acts alongside vulnerabilities, including socio-economic factors, which influence the safety of sex workers. Large international sporting events have been shown to significantly impact the economies of host countries, but there is a dearth of studies that examine how major sporting events may affect the economics of sex work and the risks taken by sex workers and clients. This study examines the determinants of the price of commercial sex alongside the price premium for and correlates of, condomless sex before, during and after the 2010 world cup in South Africa. We analysed data from three phases of repeated cross-sectional surveys with sex workers. Bivariate and multivariable logistic regression models were fitted to examine the predictors of condomless sex. We also fitted fixed-effect regression models to examine the determinants of the price of commercial sex across each survey phase. Findings suggest that the price of sex was higher during the world cup compared to before and after, whilst the price premium for condomless-sex increased from 36% before the world cup to 40% (p-value<0.001) and 57% (p-value<0.001) during and after the world cup, respectively. Across the survey phases, anal, oral or masturbation sex were more likely to be supplied without a condom compared to vaginal sex. The type of sex was the primary determinant of the price of sex across all phases. We show indicative evidence that the 2010 world cup was associated with an increase in the price of sex and supply of condomless-sex. Although these findings should be interpreted as associations rather than causal relationships, we recommend that countries with substantial sex-worker populations that host major events shouldexplicitly consider the context and structures of sex work, and promote client-focused safe-sex-interventions that explicitly consider the economic pressures faced by sexworkers to provide riskier acts, to minimise health impacts.

## 1 Introduction

Globally, sex workers (SWs) have high risks for acquiring HIV and other sexually transmitted infections (Pawa et al., 2013; Bekker et al., 2015) and experiencing violence (Deering et al., 2014a). For instance, female SWs (FSWs) in low- and middle-income countries are 13.5 times more likely to be HIV positive than women aged 15–49 years in the general population (Baral et al., 2012). Besides, FSWs face substantial structural barriers to managing risk in commercial acts, including client resistance to condom use, threats and use of violence by clients and police, a criminalised environment and strong competition from other FSWs (Beattie et al., 2010; Deering et al., 2014b; Pronyk et al.; Rao et al., 2010; Wojcicki & Malala, 2001). Besides the poverty level of SWs (especially being the poorest), financial incentives to supply condomless or otherwise riskier acts have been shown to influence FSW choices (Cunningham & Kendall, 2011; Gertler et al., 2005; Quaife, Vickerman, Manian, Eakle, Cabrera-Escobar, et al., 2018).

Major sporting events, such as the FIFA World Cup (WC) can lead to large, temporary influxes of visitors to host countries. For instance, it was estimated that around 300,000 people visited South Africa between June and July 2010 for the WC (FIFA, 2010). Visitor numbers were even higher in the 2014 WC in Brazil and the 2018 WC in Russia where over 5 million people visited WC hosting cities (FIFA, 2014; Tass, 2018). Positive impacts related to the WC have been reported, ranging from an improvement in tourism (Allmers & Maennig, 2009), trade flows (Lamla et al., 2014), and a positive impact on the stock market (Ramdas et al., 2015) to the excitement for the event among people in the host country (Allmers & Maennig, 2009). Despite these, such events can be seen as a shock to the market for commercial sex, thus increasing the demand for sex work, potentially changing prices, pressures, and incentives for SWs, including whether to supply condomless sex. Previous studies on the economics of sex work during major sporting events (Lindenblatt, 2015; Richter, Luchters, et al., 2012) found slight increases in the supply and demand for commercial sex, including a rise in the price of paid sex during the European Championship (Lindenblatt, 2015). Other studies have looked at other exogenous shocks to the commercial sex market: Cunningham and Kendall (2011) reported a 40% rise in sex work adverts on classified websites during political conventions in the US, whilst FSWs were willing to supply riskier sex during the 2007 post-election crisis in Kenya than before the crisis (Dupas & Robinson, 2012).

Several factors may explain the price of sex and the presence of a price premium for condomless-sex (Elmes et al., 2014; Gertler et al., 2005; Jakubowski et al., 2016; Manda, 2013; Quaife et al., 2019; Rao et al., 2003), including 1) extreme financial need; 2) the preferences of and demands by clients; 3) bribery and violence by police officers where especially where sex-work is criminalised, as in South Africa; 4) extreme power differentials and the subsequent possibility of violence between the SW and their clients, especially for street-based SWs; and 5) the stigmatisation of sex work (Wojcicki & Malala, 2001; Richter et al., 2013b). Furthermore, empirical evidence indicates that price premiums, also known as *condom differential*, may reduce FSW ability to negotiate for clients to use condoms or other protective strategies (Gertler et al., 2005; Ntumbanzondo et al., 2006; Rao et al., 2003). The premium is a compensating differential for the disutility, STI risk and/or pregnancy risk that SWs face when supplying condomless sex (Manda, 2013; Gertler et al., 2005; Quaife, Vickerman, Manian, Eakle, Cabrera-Escobar, et al., 2018Quaife, Vickerman, Manian, Eakle, Cabrera‐Escobar, et al., 2018; Rao et al., 2003). The price of condomless sex has been modelled as determined by a client's willingness to use condoms, a SW's insistence on condom use, and their respective bargaining power.

However, this evidence has mostly focussed on FSWs, not in the context of international sporting events or other exogenous shocks and not in a country with a high prevalence of HIV as were in South Africa (Elmes et al., 2014; Goldenberg et al., 2010; Jakubowski et al., 2016; Gertler et al., 2005; Islam & Smyth, 2012). This study fills these gaps by examining the price and determinants of the price of sex, the price premium of condomless sex compared to condom-protected sex, and determinants of the supply of unprotected sex before, during and after the 2010 WC in South Africa.

## 2 Methods

### 2.1 Study overview

This is a secondary analysis of data collected in 2010, from three cities in South Africa that hosted matches during the 2010 FIFA WC – 1) Hillbrow and Sandton in Johannesburg, 2) Salt River and Wynberg in Cape Town, and 3) Rustenburg (Richter et al., 2012a, 2013a). These cities were chosen as SWs could be reached through the sex-work organisations *Sisonke* and the *SW Education & Advocacy Taskforce* (SWEAT) (Sisonke, 2010; Sweat, 2010) or researchers in these sites (Nyangairi, 2010; Williams et al., 2003).

Data were collected from three phases of repeated cross-sectional surveys in 2010 (before the WC – April, and May; during the WC – June and July; and after the WC – August and September) with self-identified SWs. Prior to the data collection, the SW organisations identified peer educators who were SWs in the selected cities and who were purposefully selected based on previous engagement. These peer educators were then trained as SW research assistants. The research assistants then administered questionnaires to every third individual they identified as a SW. Ethics approval was provided by the University of the Witwatersrand Human Research Ethics Committee and the Research Ethics Committee at LSHTM. Respondents participated voluntarily and gave written informed consent.

### 2.2 Analysis

#### 2.2.1 Study measures

A SW was the unit of these analyses. Each SW interview elicited data on two sex acts (the most recent and the second most recent acts). We examined: 1) the price of sex - the average amount of money a SW was paid for a sex act in the two sex acts, 2) the price premium – the extra price a SW charged for condomless-sex compared to the price of protected sex (Rao et al., 2003), and 3) whether or not a SW supplied condomless-sex. Several measures were computed across each of the survey phases. First, we computed the mean price of sex for any type of sex and by condom-use status. Second, we computed the proportion of sex acts where a condom was used. Unpaired Student's t-tests were used to assess any substantive differences in the mean prices of sex and prevalence of condom use by making mean comparisons of phases 1:2, 2:3 and 1:3. Third, we computed both absolute price premiums (differences in the price of protected sex and condomless sex) and relative price premium (ratio between price premium of unprotected sex to protected sex) within each survey phase.

#### 2.2.2 Unadjusted and adjusted logistic regression estimation

Two steps were adopted in these analyses. First, bivariate analyses were performed using logistic regression to identify factors independently associated with the supply of unprotected sex. A multicollinearity check using Pearson's R correlation was performed applying a threshold of r ≥ 0.7 as the cutoff point (Katz, 2011; Mukaka, 2012). In each survey phase, all covariates were then incorporated into a second step. Second, we fitted multivariable logistic regression models for each phase. In these models, the dependent variable was the self-reported supply of condomless sex in either of the last two recent sex acts. The inclusion of all variables irrespective of their strength of association with the supply of condomless sex from the bivariate analysis was to make inferences about how the WC may change the determinants of the supply of condomless sex. We included the price of sex (natural log) (Quaife et al., 2019), the gender of a SW, the level of education (Elmes et al., 2014; Rao et al., 2003), the place where sex work is solicited such as street or hotel or in a massage parlour (Adriaenssens & Hendrickx, 2012), the type of sex supplied (Baral et al., 2012), whether a SW has a spouse, girlfriend or boyfriend, whether the SW had an alternative source of income, their age, whether the SW was drunk during sex, whether the SW had contact with police in the past year, the number of dependents (children or adults), whether the SW visited a hospital in the month preceding the survey and their occupation before sex work. These factors have been associated with the supply of condomless-sex elsewhere (Adriaenssens & Hendrickx, 2012; Arunachalam & Shah, 2013; Elmes et al., 2014; Shannon et al., 2015; Wojcicki & Malala, 2001). We did not assess clients' role in the transaction and the degree to which they influenced the use of condom and how they did as data was unavailable.

#### 2.2.3 Fixed effects models

Where we had data on more than one act per SW (i.e. within survey rounds), sex-worker fixed-effect models were useful to explore the determinants of the price of sex in the market for sex (Adriaenssens & Hendrickx, 2012; Arunachalam & Shah, 2013; Gertler et al., 2005; Manda, 2013; Muravyev & Talavera, 2018; Robinson & Yeh, 2011). In this dataset, the repeated cross-section design means that we cannot use fixed-effects models to explore heterogeneity across rounds. We employed SW fixed effects models due to their ability to control for unobserved heterogeneity and subsequent omitted variable bias (Hedges, 1994). The coefficient from the fixed effects models represents the percentage (%) change in the geometric mean price of sex associated with a unit increase for continuous predictors or being in a level ‘*b’* relative to level ‘*a’* for binary or categorical variables. The % change is calculated by subtracting 1 from the exponent of the coefficient, that is:

% Change = (exp(coefficient) – 1) * 100

To examine whether random-effects models would have been the most appropriate models as opposed to fixed effects models, Hausman Tests were performed in each of the three survey phases (Baltagi, 2008; Hausman, 1978).

## 3 Results

### 3.1 Sample characteristics

Table 1 shows the distribution of the study sample over the three survey phases (before, during and after the 2010 WC in South Africa). A total of 2260 SWs (671 before the WC, 781 during, and 808 after the WC) were surveyed. Female SWs were the majority of respondents across all three phases. Most SWs worked from the street and hotel/brothel before and after the WC while a majority of SWs worked in a combination of places (street, hotel/brothel, massage parlour or shebeen) during the WC. SWs surveyed after the WC were significantly more likely to have been jobless prior to sex work than both before (p-value<0.015) and during (p-value<0.037) the WC. The non-commercial partner rate of SWs after the WC (38.4% [95% CI 35.1–41.8]) was significantly higher than before (30.0% [95% CI 27.1–34.0; p-value = 0.002]) and during (32.0% [95% CI 28.8–35.4; p-value = 0.008]) the WC. Overall, the average age of SWs was 29.9 [95% CI 29.7–30.2] years with 31.6% [95% CI 29.6–33.6] of SWs having at least secondary education while 70.1% [95% CI 68.1–72.0] did not have an alternative source of income and had served an average of 15 [95% CI 15–16] clients in the week preceding the survey.

**Table 1:** Distribution of sample before, during and after the 2010 WC by selected sociodemographic factors.

	Before the WC		During the WC		After the WC		Overall	
	Total number	%/(Mean)/[Median]	Total number	%/(Mean)/[Median]	Total number	%/(Mean)/[Median]	Total number	%/(Mean)/[Median]
**Age**	668	(30.2) [30]	775	(29.9) [29]	795	(29.7) [29]	2238	(29.9) [29]
**Gender**								
Female	600	92.6	689	91.0	715	92.3	2004	91.9
Male	34	5.3	40	5.3^‡^	23	3.0^§^	97	4.5
Transgender	14	2.12	28	3.7	37	4.8^§^	79	3.6
**Education**								
No education	133	20.9	127	17.0	152	19.9	412	19.2
Primary	319	50.2	382	51.2	354	46.4	1055	49.2
Secondary+	184	28.9	237	31.8	257	33.7	678	31.6
**Place of sex work**								
Street	225	38.6^†^	231	32.4	220	29.7^§^	676	33.2
Hotel/Brothel	202	34.7^†^	183	25.7^‡^	281	37.9	666	
Massage	6	1.0	7	1.0	14	1.9	27	1.3
Shebeen	74	12.7^†^	41	5.8	44	5.9^§^	159	7.8
Combination	76	13.0^†^	250	35.1^‡^	183	24.7^§^	509	25.0
**Previous work before sex work**								
No Job	149	30.4	196	31.7^‡^	241	37.3^§^	586	33.4
Cashier	40	8.2	71	11.5	68	10.5	179	10.2
Beauty therapist	74	15.1	108	17.5	104	16.1	286	16.3
Seamstress/Tailor	35	7.1	29	4.7	29	4.5	93	5.3
Student	118	24.1	129	20.9	111	17.2^§^	358	20.4
Waitress	74	15.1	85	13.6	93	14.4	252	14.4
**Alternative income**								
No	480	75.5	519	71.5^‡^	487	64.2^§^	1486	70.1
Yes	156	24.5	207	28.5^‡^	272	35.8^§^	635	29.9
**Has a Partner**								
No	464	69.6	531	68.0^‡^	488	61.6^§^	1483	66.2
Yes	203	30.4	250	32.0^‡^	304	38.4^§^	757	33.8
**Number of clients in the last week**	670	(16) [10]	778	(14) [10] ^‡^	808	(16) [12]	2256	(15) [11]
**Number of dependents**								
None	77	11.5	82	10.5	103	12.8	262	11.6
1 to 3 adults or children	359	53.5	477	61.1	464	57.4	1300	57.5
4 or more adults or children	235	35.0	222	28.4	241	29.8	698	30.9
**Whether had contact with the police in the past year**								
No	454	70.5	511	70.9	541	70.4	1506	70.6
Yes	190	29.5	210	29.1	227	29.6	627	29.4
**Whether visited a hospital in the last month**								
No	252	39.6^†^	337	46.2	295	39.6^§^	884	41.9
Yes	385	60.4^†^	392	53.8	450	60.4^§^	1227	58.1

† Significant differences (p-value<0.05) Phase 1:2.

‡ Significant differences (p-value<0.05) Phase 2:3.

§ Significant differences (p-value<0.05) Phase 1:3.

### 3.2 Price of sex, price premiums, and prevalence of condom use

**Fig. 1:**
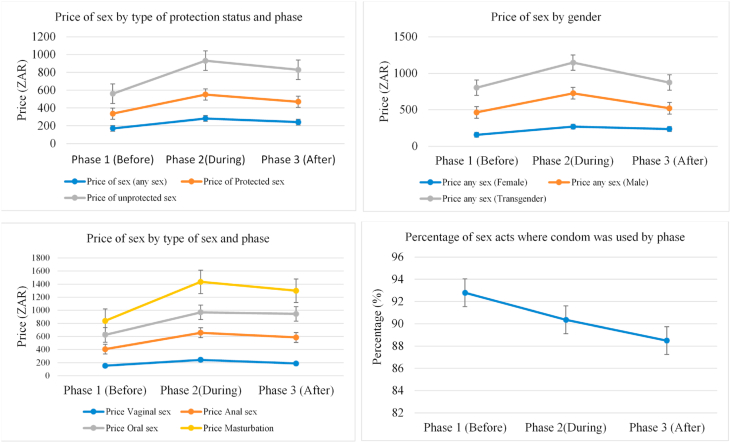
Price of by protection status, gender and type of sex and the prevalence of condom use in each of the survey phases.

Fig. 1 shows the mean prices of sex and the proportion of sex acts where a condom was used among SWs in their two most recent sex acts. On average, SWs charged ZAR 281.5 [95% CI: 259.3–303.6] for any sex (protected or unprotected) during the WC which was significantly higher than the price charged before (ZAR 170.3 [95% CI: 160.9–179.8; p-value<0.001]) and after the WC (ZAR 241.6 [95% CI: 224.6–258.7; p-value = 0.005]).

Transgender SWs charged higher prices, except in phase 2 where male SWs charged more (ZAR 457.1 [95% CI: 352.0–562.1]). The price of any sex among female and male SWs was significantly higher during the WC compared to before (p-value <0 0.001 and p-value = 0.018) and after (p-value = 0.034 and p-value = 0.020) respectively.

Four types of sex were supplied – vaginal, anal, oral, and masturbation sex. Notably, the price of each respective type of sex was significantly (p-value < 0.05) higher during the WC than before the WC. In absolute terms and compared to the price of the other types of sex in each phase, anal sex was more priced before and after the WC where SWs charged ZAR 252.9 [95% CI: 211.1–294.7] and ZAR 397.6 [95% CI: 289.9–505.3] respectively, however, masturbation sex was more costly during the WC at an average of ZAR 465.6 [95% CI: 358.6–572.7].

The price of condomless-sex was significantly higher than the price of protected sex in all survey phases (Fig. 1). For instance in phase 1, unprotected sex on average cost ZAR 58.8 [95% CI: 22.8–94.8, p-value = 0.0014] more than protected sex (mean ZAR 165.8 [95% CI: 156.3–175.2], representing a price premium of 35.5% [95% CI: 8.6–62.4]. This premium increased to 40.4% [95% CI: 1.8–78.9] representing a price increase of ZAR 109.1 [95% CI 33.1–185.1, p-value = 0.005]) in phase 2, and in phase 3 reached the highest premium of 56.9% [95% CI: 20.2–93.6] representing a price increase of ZAR 130.1 [95% CI 75.4–184.9, p-value<0.001]).

Condom use was highest in phase 1 (92.8%) but reduced by 2.4 percentage points (p-value = 0.021) to 90.4% in phase 2 with a further 1.9 percentage point (p-value = 0.093) reduction in condom use between phase 2 and phase 3 (Fig. 1).

### 3.3 Correlates of unprotected/condomless-sex

#### 3.3.1 Bivariate analysis

Across all phases, the price of sex, being of the male gender or transgender (except phase 3) compared to being female, supplying anal, oral or masturbation sex compared to vaginal sex and a SW being drunk during sex were all significantly (at the 5% level) associated with an increase in the odds of supplying unprotected sex (Table 2). Although age was a marginally significant predictor in phase 1 (p-value = 0.053) and not related in phase 2 (p-value = 0.985), a one-year increase in the SW's age was significantly associated with a 5% [OR = 0.95 (95% CI 0.93–0.98, p-value = 0.001)] decrease in the odds of supplying condomless-sex. The number of clients seen by a SW was also an important predictor in bivariate analysis. A unit increase in the number of clients seen in the week preceding the survey was significantly associated with a 27% (p-value = 0.004) and 34% (p-value<0.001) reduction in the odds of supplying unprotected sex before and after the WC respectively. Further bivariate analyses revealed that SWs had significantly 27% [OR = 0.73 (95% CI 0.56–0.95, p-value = 0.022)] reduced odds of supplying condomless sex before the WC compared to during the WC. In similar comparisons between during and after the WC, no significant evidence of increased odds of supplying condomless sex was observed [OR = 1.22 (95% CI 0.97–1,54, p-value = 0.093)].

**Table 2:** Unadjusted odds ratios (OR), 95% confidence intervals and p-values for the correlates of the supply of unprotected sex among SWs before, during and after the 2010 WC in South Africa.

Dependent variable: supply of condomless-sex in previous 2 acts	Before (Phase 1)		During (Phase 2)		After (Phase 3)	
	Unadjusted Odds ratio [95% CI]	p-value	Unadjusted Odds ratio [95% CI]	p-value	Unadjusted Odds ratio [95% CI]	p-value
**Price (ln)**	1.6 [1.3–2.1]	<0.001	1.3 [1.0–1.5]	0.021	1.3 [1.1–1.6]	0.005
**Gender**						
Female						
Male	5.6 [2.8–10.9]	<0.001	5.2 [2.7–10.2]	<0.001	4.0 [1.7–9.3]	0.001
Transgender	4.6 [1.7–12.5]	0.003	4.4 [2.3–8.5]	<0.001	1.6 [0.8–3.2]	0.230
**Level of Education**						
None						
Primary	0.4 [0.2–0.8]	0.013	0.8 [0.5–1.4]	0.481	1.0 [0.6–1.7]	0.961
Secondary+	1.0 [0.5–2.0]	0.993	1.2 [0.7–2.2]	0.492	1.2 [0.7–2.1]	0.449
**Location of sex work**						
Street						
Hotel/Brothel	0.7 [0.4–1.4]	0.284	0.4 [0.2–0.7]	0.003	1.0 [0.6–1.6]	0.956
Massage	4.9 [0.9–26.7]	0.066	0.6 [0.1–4.6]	0.656	0.4 [0.1–2.6]	0.299
Shebeen	0.9 [0.4–2.2]	0.801	2.0 [0.9–4.4]	0.084	2.0 [0.9–4.3]	0.070
Combination	2.2 [1.0–4.6]	0.041	1.0 [0.6–1.6]	0.908	1.6 [1.0–2.6]	0.054
**Age (Years)**	1.0 [0.9–1.0]	0.053	1.0 [1.0–1.0]	0.985	1.0 [0.9–1.0]	0.001
**Previous work**						
No job						
Cashier	0.6 [0.2–2.2]	0.442	0.7 [0.3–1.7]	0.471	0.5 [0.2–1.0]	0.056
Beauty/hairdresser	1.8 [0.8–4.2]	0.189	0.8 [0.4–1.6]	0.534	1.1 [0.6–1.9]	0.765
Tailor	0.7 [0.1–3.5]	0.671	1.1 [0.4–3.1]	0.879	0.8 [0.3–2.5]	0.719
Student	1.1 [0.5–2.5]	0.762	0.8 [0.5–1.5]	0.524	1.3 [0.8–2.2]	0.364
Waitress	1.1 [0.4–3.0]	0.811	0.6 [0.3–1.3]	0.199	1.4 [0.8–2.6]	0.227
**Alternative income source**						
No						
Yes	1.2 [0.7–2.1]	0.435	1.08 [0.7–1.7]	0.740	1.0 [0.7–1.5]	0.954
**Has a non-commercial partner**						
No						
Yes	0.9 [0.5–1.5]	0.606	0.70 [0.5–1.1]	0.099	1.3 [0.9–1.8]	0.224
**Number of clients seen in the last week (ln)**	0.7 [0.6–0.9]	0.004	1.11 [0.9–1.4]	0.329	0.7 [0.5–0.8]	<0.001
**Type of sex**						
Vaginal sex						
Anal sex	8.1 [4.3–15.2]	<0.001	12.8 [7.0–23.1]	<0.001	5.6 [3.3–9.6]	<0.001
Oral sex	8.5 [4.4–16.3]	<0.001	8.0 [4.5–14.3]	<0.001	6.7 [4.4–10.4]	<0.001
Masturbation	13.0 [5.7–29.5]	<0.001	21.7 [11.3–41.7]	<0.001	12.4 [6.8–22.8]	<0.001
**Whether drunk during sex**						
No						
Yes	1.63 [1.01–2.63]	0.046	1.94 [1.30–2.90]	0.001	2.03 [1.45–2.85]	<0.001
**Number of dependents**						
None						
1 to 3 adults or children	0.37 [0.19–0.74	0.005	0.62 [0.34–1.13]	0.118	0.56 [0.35–0.90]	0.017
4 or more adults or children	0.49 [0.24–0.99	0.045	0.60 [0.31–1.19]	0.143	0.41 [0.24–0.72]	0.002
**Whether had contact with the police in the past year**						
No						
Yes	0.89 [0.51–1.56]	0.692	1.16 [0.75–1.80]	0.510	0.94 [0.63–1.40]	0.747
**Whether visited a hospital in the last month**						
No						
Yes	0.73 [0.44–1.20]	0.215	0.78 [0.52–1.18]	0.241	0.67 [0.46–0.97]	0.033

Notably, the independent effects of gender and having an alternative source of income on the supply of condomless sex decreases from phase 1 to phase 3. On the contrary, being drunk during sex became more important from phase 1 to phase 3 where SWs had 63% (p-value = 0.046), 94% (p-value = 0.001) and 103% (p-value<0.001) increased odds of supplying condomless-sex compared to their counterparts who were not drunk before, during and after the WC respectively.

#### 3.3.2 Multivariable logistic regression estimation

Table 3 shows the adjusted odds ratios (AOR) of the correlates of the supply of condomless sex before, during and after the 2010 WC in South Africa. After controlling for other factors, the price of sex was no longer a significant determinant of the supply of condomless sex across all survey phases unlike in bivariate analyses. Like the bivariate analyses, only the type of sex (anal, oral and masturbation compared to vaginal) was consistently a significant positive predictor of the supply of condomless sex in models of before (except masturbation, p-value = 0.183), during and after the WC. In phase 1, age and the type of sex were the significant determinants of the supply of condomless sex. A one-year increase in a SW's age was associated with a 10% (p-value = 0.001) reduction in the odds of supplying condomless sex. In terms of the type of sex, compared to SWs who supplied vaginal sex, SWs who supplied anal or oral sex had 54.9 times (p-value<0.001) and 23.2 times (p-value<0.001) the odds of supplying condomless sex. Unlike in phase 1, relative to supplying vaginal sex, SWs were significantly more likely to supply masturbation sex in phases 2 and 3. Although not expected, relative to SWs without an alternative source of income, SWs with an alternative source of income had 2.2 times [95% CI 1.0–4.7, p-value = 0.039] the odds of supplying condomless sex after controlling for other factors in Phase 2.

**Table 3:** Adjusted odds ratios (AOR), 95% confidence intervals and p-values for determinants of supply of unprotected sex among SWs before, during and after the 2010 WC in South Africa.

	Before (Phase 1)		During (Phase 2)		After (Phase 3)	
	Adjusted Odds ratio [95% CI]	p-value	Adjusted Odds ratio [95% CI]	p-value	Adjusted Odds ratio [95% CI]	p-value
**Price (ln)**	1.3[0.6–2.6]	0.530	0.9[0.6–1.2]	0.407	0.9[0.6–1.2]	0.396
**Gender**						
Female	Ref		Ref		Ref	
Male	1.1[0.2–6.2]	0.927	0.8[0.2–3.1]	0.797	0.7[0.2–3.5]	0.708
Transgender	0.1[0.0–0.6]	0.012	0.9[0.3–2.8]	0.917	0.9[0.3–3.1]	0.867
**Level of Education**						
None	Ref		Ref		Ref	
Primary	0.7[0.1–5.8]	0.761	0.6[0.2–1.5]	0.252	0.9[0.4–2.2]	0.844
Secondary+	1.7[0.2–12.5]	0.601	0.6[0.2–1.5]	0.265	0.8[0.4–2.0]	0.714
**Location of sex work**						
Street	Ref		Ref		Ref	
Hotel/Brothel	0.9[0.2–3.7]	0.865	0.4[0.2–1.1]	0.069	0.6[0.3–1.2]	0.122
Massage	5.0[0.1–300.9]	0.439	10.1[2.1–48.7]	0.004		
Shebeen	2.9[0.4–20.5]	0.275	1.1[0.2–5.0]	0.950	2.4[0.6–8.8]	0.202
Combination	1.1[0.2–7.0]	0.947	0.6[0.2–1.3]	0.160	0.5[0.2–1.1]	0.082
**Age (Years)**	0.9[0.8–0.9]	0.001	1.0[0.9–1.1]	0.885	1.0[0.9–1.0]	0.277
**Previous work**						
No job	Ref		Ref		Ref	
Cashier	1.2[0.2–7.5]	0.833	0.8[0.2–2.4]	0.638	0.5[0.2–1.4]	0.178
Beauty/hairdresser	0.2[0.0–2.1]	0.167	0.4[0.1–1.1]	0.071	1.0[0.4–2.2]	0.913
Tailor	2.7[0.4–19.8]	0.323	0.9[0.3–3.2]	0.923	1.2[0.2–6.5]	0.799
Student	1.2[0.4–4.2]	0.733	0.8[0.3–2.0]	0.614	1.0[0.4–2.2]	0.932
Waitress	1.5[0.2–12.2]	0.723	0.5[0.2–1.6]	0.242	0.8[0.3–2.2]	0.666
**Alternative income source**						
No	Ref		Ref		Ref	
Yes	0.3[0.1–1.6]	0.168	2.2[1.0–4.7]	0.039	0.9[0.5–1.9]	0.844
**Has a non-commercial partner**						
No	Ref		Ref		Ref	
Yes	0.7[0.2–2.4]	0.592	0.5[0.2–1.1]	0.070	1.3[0.8–2.4]	0.305
**Number of clients seen in the last week (ln)**	0.7[0.4–1.3]	0.303	1.4[0.8–2.5]	0.223	0.8[0.6–1.2]	0.265
**Type of sex**						
Vaginal sex	Ref		Ref		Ref	
Anal sex	54.9[15.0–200.4]	<0.001	10.5[4.3–25.4]	<0.001	5.5[2.1–14.9]	0.001
Oral sex	23.2[6.6–81.6]	<0.001	20.1[8.2–49.1]	<0.001	14.5[7.2–28.8]	<0.001
Masturbation	8.0[0.4–169.2]	0.183	33.5[13.1–86.1]	<0.001	33.1[13.4–82.2]	<0.001
**Whether drunk during sex**						
No	Ref		Ref		Ref	
Yes	1.0[0.3–3.8]	0.959	1.6[0.8–3.2]	0.161	3.3[1.9–5.8]	<0.001
**Number of dependents**						
None	Ref		Ref		Ref	
1 to 3 adults or children	3.3[0.8–14.2]	0.111	1.3[0.5–3.9]	0.576	0.4[0.2–0.9]	0.035
4 or more adults or children	4.0[0.7–21.9]	0.109	0.9[0.3–2.9]	0.807	0.4[0.1–1.1]	0.088
**Whether had contact with the police in the past year**						
No	Ref		Ref		Ref	
Ref	0.7[0.2–2.2]	0.514	0.6[0.3–1.1]	0.110	0.9[0.5–1.7]	0.705
**Whether visited a hospital in the last month**						
No	Ref		Ref		Ref	
Yes	1.4[0.4–5.1]	0.631	0.7[0.4–1.4]	0.357	0.9[0.4–1.7]	0.649
**Log pseudolikelihood**	-82.3		-192.3		-212.2	
**Pseudo r-Squared**	0.4		0.3		0.3	
**Number of obs**	661		847		836	

Ref – Reference category; -Excluded from the model; ln – Natural logarithm.

In phase 3, however, in addition to the type of sex, a SW being drunk during sex [AOR = 3.3 (95% CI: 1.9–5.8), p-value<0.001] was associated with increased odds of supplying condomless sex. However, SWs with 1–3 dependents (children or adults) were significantly associated with a 60% reduction [AOR = 0.4 (95% CI: 0.2–0.9), p-value = 0.035] in their odds of supplying condomless-sex compared to SWs without dependants even though 70.8% them had no alternative source of income.

#### 3.3.3 Determinants of the price of sex

Table 4 shows the coefficients from multivariable fixed-effects and random-effects models for regressions of the price of sex and independent variables in phases 1, 2 and 3. In the fixed-effects models, condom use was not a consistent predictor of the price of sex as it appeared to reduce the price of sex by 6.9% (p-value = 0.492) in phase 1 but increase the price by 9.9% (p-value = 0.323) in phase 2 and 25.7% (p-value = 0.021) phase 3. Compared to vaginal sex, only anal sex was a consistent significant positive predictor of price across all three survey phases. The price of sex was 24.8% (p-value = 0.017), 28.2% (p-value = 0.002) and 46.8% (p-value = 0.001) higher for anal sex than for vaginal sex before, during and after the WC respectively.

**Table 4:** Multivariable fixed effects and random effects models showing the regression of price received by SWs before (Phase 1), during (Phase 2) and after (Phase 3) the 2010 WC in South Africa.

Dependent variable: ln price of previous 2 acts	Phase 1 (Before)		Phase 2 (During)		Phase 3 (After)	
	Fixed-effects	Random effects	Fixed-effects	Random effects	Fixed-effects	Random effects
**Whether condom was used**						
No	Ref	Ref	Ref	Ref	Ref	Ref
Yes	-0.1 [-0.3 to 0.1]	-0.1 [-0.4 to 0.2]	0.1 [-0.1 to 0.3]	0.1 [-0.1 to 0.3]	0.2* [0.0 to 0.4]	0.1 [-0.1 to 0.4]
**Type of sex**						
Vaginal sex	Ref	Ref	Ref	Ref	Ref	Ref
Anal sex	0.2** [0.0 to 0.4]	0.4** [0.1 to 0.6]	0.3** [0.1 to 0.4]	0.3** [0.1 to 0.5]	0.4** [0.2 to 0.6]	0.5*** [0.3 to 0.7]
Oral sex	0.3*** [0.2 to 0.5]	0.4*** [0.2 to 0.6]	0.0 [-0.1 to 0.1]	0.1 [0.0 to 0.3]	0.3*** [0.1 to 0.4]	0.4*** [0.2 to 0.5]
Masturbation	0.3* [0.0 to 0.5]	0.4 [0.0 to 0.8]	-0.1 [-0.4 to 0.1]	0.2 [-0.1 to 0.5]	0.1 [-0.2 to 0.4]	0.3 [0.0 to 0.6]
**Whether drunk during sex**						
No	Ref	Ref	Ref	Ref	Ref	Ref
Yes	0.1 [-0.1 to 0.2]	0.2** [0.1 to 0.3]	-0.0 [-0.2 - 0.2]	0.1 [0.0 to 0.3]	0.1 [-0.1 to 0.2]	0.3*** [0.1 to 0.4]
**Age**	–	0.0** [0.0 to 0.0]	–	0.0 [0.0 to 0.0]	–	0.0* [0.0 to 0.0]
**Ln number of clients seen in the last week**		-0.1** [-0.2 to 0.0]	–	-0.1** [-0.2 to 0.0]	–	-0.1** [-0.2 to 0.0]
**Gender**						
Female	–	Ref	–	Ref	–	Ref
Male	–	0.1 [-0.4 to 0.5]	–	0.4 [-0.1 to 0.9]	–	0.1 [-0.2 to 0.5]
Transgender	–	0.2 [-0.3 to 0.6]	–	0.5** [0.1 to 0.8]	–	0.2 [-0.1 to 0.5]
**Level of education**						
None	–	Ref	–	Ref	–	Ref
Primary	–	-0.1 [-0.3 to 0.1]	–	-0.2 [-0.5 to 0.1]	–	-0.1 [-.03 to 0.1]
Secondary+	–	(0.2 [-0.1 to 0.4]	–	(0.2 [-0.1 to 0.5]	–	(0.0 [-0.2 to 0.2]
**Place of Sex work**						
Street	–	Ref	–	Ref	–	Ref
Hotel/Brothel	–	0.0 [-0.1 to 0.2]	–	-0.2 [-0.4 to 0.1]	–	0.1 [-0.1 to 0.3]
Massage	–	0.2 [-0.8 to 1.3]	–	-0.3 [-0.6 to 0.1]	–	(0.0 [-0.7 to 0.7]
Shebeen	–	(0.0 [-0.2 to 0.2]	–	-0.3* [-0.6 to -0.1]	–	-0.2 [-0.4 to 0.1]
Combination^a^	–	0.2 [0.0 to 0.5]	–	(0.1 [-0.1 to 0.3]	–	(0.0 [-0.2 to 0.2]
**Previous work prior to sex work**						
No job	–	Ref	–	Ref	–	Ref
Cashier	–	0.2 [-0.1 to 0.5]	–	-0.3* [-0.6 to -0.1]	–	-0.3* [-0.5 to 0.0]
Beauty/hairdresser	–	-0.1 [-0.4 to 0.1]	–	0.0 [-0.3 to 0.3]	–	0.0 [-0.2 to 0.2]
Seamstress/tailor	–	0.0 [-0.3 to 0.3]	–	-0.2 [-0.7 to 0.3]	–	-0.2 [-0.5 to 0.1]
Student	–	-0.1 [-0.3 to 0.1]	–	-0.3** [-0.5 to -0.1]	–	-0.1 [-0.3 to 0.2]
Waitress	–	0.0 [-0.3 to 0.2]	–	-0.2 [-0.5 to 0.1]	–	-0.2 [-0.4 to 0.0]
**Has alternative income**						
No	–	Ref	–	Ref	–	Ref
Yes	–	0.1 [0.0 to 0.3]	–	0.2 [0.0 to 0.4]	–	0.0 [-0.1 to 0.2]
**Has a partner**						
No	–	Ref	–	Ref	–	Ref
Yes	–	0.0 [-0.2 to 0.2]	–	0.0 [-0.2 to 0.2]	–	0.1 [-0.1 to 0.3]
**Number of dependents**						
None		Ref	–	Ref	–	Ref
1 to 3 adults or children	–	0.1 [-0.2 to 0.4]	–	0.2 [0.0 to 0.4]	–	0.0 [-0.2 to 0.3]
4 or more adults or children	–	-0.1 [-0.4 to 0.2]	–	0.3 [0.0 to 0.5]	–	-0.1 [-0.4 to 0.1]
**Whether had contact with the police in the past year**						
No	–	Ref	–	Ref	–	Ref
Yes	–	0.3** [0.1 to 0.5]	–	-0.2* [-0.4 to 0.0]	–	0.0 [-0.2 to 0.1]
**Whether visited a hospital in the last month**						
No	–	Ref	–	Ref	–	Ref
Yes	–	-0.3*** [-0.5 to -0.2]	–	-0.2 [-0.3 to 0.0]	–	0.0 [-0.2 to 0.1]
**Number of obs.**	1267	661	1396	847	1470	848
**AIC**	790.3	n/a	900.7	n/a	1310.8	n/a
**BIC**	816.0	n/a	926.9	n/a	1337.3	n/a

Positive coefficients indicate the correlate is positively associated with the price, whereas negative coefficients indicate negative correlations.

AIC - Akaike information criterion; BIC - Bayesian information criterion; Ref – Reference; -Variable omitted; ln– Natural logarithm; n/a – not applicable.

*p-value<0.05; **p-value<0.01; ***p-value<0.001.

a “Combination” refers to SWs who worked in more than one location during their last two sex acts.

When all covariates were considered in random-effects models, in phase 1, the type of sex (anal sex – 46.4%; p-value = 0.003, and oral sex – 48.3%; p-value<0.001), a SW being drunk during sex (20.1%; p-value = 0.005), age (2.0%; p-value = 0.008) and having had a contact with the police in the past year (32.4%; p-value = 0.001) were significant positive correlates of the price of sex whereas an increase in the number of clients seen in the week preceding the survey (12.4%; p-value = 0.003) and having had a visit to a clinic in the month preceding the survey (29.5%; p-value<0.001) were negative determinants of the price of sex. In Phase 2, supplying anal sex (35.1%; p-value = 0.001) compared to vaginal sex, being a transgender SW (61.8%; p-value = 0.009) compared to being a female SW were the positive predictors of the price while holding all other variables constant, a client increase (13.1%; p-value = 0.008), working in a shebeen (28.4%; p-value = 0.017) compared to the street, having had a clinic visit in the month preceding the survey (18.6%; p-value = 0.036) and having been a cashier or student relative to having had no job prior to sex work was associated with a 28.8% (p-value = 0.018) and 26.2% (p-value = 0.007) reduction in the mean price of sex respectively.

In phase 3, the type of sex, being drunk during sex, being a transgender SW compared to a female SW and the age of the SW were the significant positive correlates of price. Similar results to the random effects models were obtained with ordinary least squares regression. In comparison to the fixed-effects model, the random-effects model was rejected across all phases (p-value = 0.019, p-value<0.001 and p-value<0.001 in the before, during and after the WC model comparisons respectively), however, we present its results for comparison purposes.

## 4 Discussion

This study examined the economics of sex work before, during and after the 2010 FIFA WC in South Africa. Specifically, we examined the levels and determinants of the price of sex and price premium of unprotected sex, alongside the determinants of the supply of condomless-sex among SWs using three waves of data collected before (phase 1), during (phase 2) and after (phase 3) the WC. We found that SWs were paid significantly more for sex during the WC than before and after, while condom use decreased. Additionally, there were significant price premiums of 35.5%, 40.4% and 56.9% before, during and after the WC respectively.

The reason for the higher prices of sex and price premiums can be hypothesised as follows. First, it was estimated that nearly 1.4 million people visited South Africa from June to July 2010 with over 300,000 having visited due to the WC (FIFA, 2010). As economic theory predicts, the convergence of a large number of people would increase demand for commercial sex, assuming the number of SWs did not increase to the same extent (Cunningham & Kendall, 2011) which would increase prices. Second, as in many other countries worldwide, sex work in South Africa is illegal and criminalised (Arnott & Crago, 2009; Richter et al., 2010). These factors influence the price formation in the market for sex. For instance, the illegality of sex work presents a barrier for SWs entering the market, thus increasing prices due to lower supply than demand (Gale, 1955, pp. 155–169).

Although price premiums obtained in this study are not as high as those reported in India (79%) (Rao et al., 2003), in Kenya (136%) (Jakubowski et al., 2016) and in the Democratic Republic of the Congo (SWs charged 350% times more for condomless-sex compared to protected sex) (Ntumbanzondo et al., 2006), they are relatively high and in the same range as those in Zimbabwe (42.9%) (Elmes et al., 2014), in Ecuador (33%) (Arunachalam & Shah, 2013) and in Mexico (23%) (Gertler et al., 2005).

Third, the price premiums reported in this study are similar to those described in the literature as condom differential, compensating differentials or risk premiums (Cunningham & Shah, 2016; Rao et al., 2003) where SWs accepted a higher price to compensate for the risks of HIV and STI infection as well as pregnancy and discomfort related to the supply of condomless-sex (Gertler et al., 2005; Adriaenssens & Hendrickx, 2012). This could increase the incidence of HIV and STIs among these SWs and their clients. Fourth, the WC can be hypothesised as a temporary positive shock to the market for sex thus increasing both the demand for sex and income of SWs during the WC. Evidence from the 2010 WC showed no significant changes to the number of new entrants to sex work and the influx of SWs into WC cities (Richter, Luchters, et al., 2012). Fifth, Rao et al. showed that SWs face potential income losses of up to 79% by not providing condomless sex (Rao et al., 2003). Financially vulnerable SWs who provide for several dependents may accept condomless sex for higher fees to avoid further impoverishment resulting from an income loss. As a result, poorer SWs will inequitably bear higher HIV, STI and pregnancy burdens compared to their better-off counterparts (Monroe, 2005; Saggurti et al., 2011). Sixth, with the premium significantly higher (p-value = 0.018) during the WC compared to before, it can be argued that SWs use the price premium to smooth supply during exogenous shocks similar to evidence presented by Robinson and Yeh (Robinson & Yeh, 2011) where SWs engaged in more risky sex to get better compensation during a health shock.

Across all three phases, relative to vaginal sex, anal sex, oral sex, and masturbation remained significant positive correlates of the supply of condomless sex. Although other studies have reported increased odds for the supply of condomless-sex for either anal or oral sex compared to vaginal sex (Schwandt et al., 2006; Pebody, 2010; Owen et al., 2019), this is the first study to quantitatively present this in the context of a major international sporting event. The high odds for these sex types may be related to financial incentives such as higher prices for these special services.

The findings on the determinants of the price of sex from this study are similar to those reported elsewhere in other studies (Elmes et al., 2014; Levitt & Venkatesh, 2007). In this study, however, age was not a significant and consistent predictor as reported in other studies where age was a significant negative predictor of the price of sex (Voeten et al., 2007). This could be a contextual difference. Again, unlike in other studies, this study found that condom use was associated with the increased price of sex but only after the WC. This was not expected, however, perhaps SWs earned more money during the WC thus reducing their need for extra cash after the WC which in effect increased their bargaining power for higher prices. Future studies should investigate this further.

Results from this study should be interpreted in light of the following limitations. First, although the price of sex and price premiums were found to be significantly higher during the WC compared to before, these findings should be interpreted as an association rather than a causal relationship. This is because the study involved a repeated cross-sectional design where different SWs were surveyed at each phase which does not permit the examination of a causal effect. Future studies could employ a panel design where the same SWs will be surveyed before, during and after the major sporting event. Similarly, researchers could establish an unexposed group of SWs (for instance, SWs in areas where WC matches – or any other major sporting event – are not played) and at the same time survey exposed SWs (SWs where WC matches are played) to examine the causal effect of the major sporting event on the prices of sex and supply of risky acts.

Second, the non-significant effect of condom use on the price of sex may be due to endogeneity. The endogeneity may have arisen due to a positive correlation between other unobserved factors such as a SW's attractiveness or bargaining power and the price of sex which may have biased the estimates in this study towards zero. To counter the endogeneity problem, two primary approaches have been applied. First, some studies have fitted models including proxy measures of the unobserved factors, for instance, the attractiveness of a SW (Islam & Smyth, 2012). Second, other studies have used instrumental variables (IV) or fixed-effects models (Adriaenssens & Hendrickx, 2012; Arunachalam & Shah, 2013; de la Torre et al., 2010; Gertler et al., 2005; Islam & Smyth, 2012; Manda, 2013; Rao et al., 2003).

Third, this study had a relatively small sample size especially of male and transgender SWs which may not be representative of the population of male and transgender SWs in South Africa. Furthermore, some SWs may work in areas not reached by this survey, for example primarily connecting to clients online, making this work less generalisable across all SWs.

As SWs and clients operate in an economic environment, policymakers must expect them to respond rationally to changes in market forces. Therefore, established interventions during major events should 1) reflect the rational adaptation to changes in market forces, 2) not judge or blame SWs for responding to market forces, 3) should consider adopting structural changes such as law reform, and 4) consider the specific needs of high-risk groups such as protection from abuse by police and clients and clamp down when events are held in areas where sex work is common, or where HIV or other STI prevalence is high to mitigate infection risks.

## 5 Conclusion

There is limited evidence on how major sporting events impact the price of sex and the riskiness of sex acts among SWs especially in countries with high HIV prevalence. We examined how the 2010 FIFA WC influenced the price of sex, the supply of paid condomless sex and their determinants. We show that the WC was associated with an increase in the price of sex, price premiums, and the supply of condomless sex. These findings may imply a possible increase in the transmission of HIV and other STIs especially when such events are held in settings with a high SW population and/or high HIV and STI prevalence. Therefore, countries planning major sporting events especially in such settings should consider adopting structural changes such as law reform (e.g. legalizing sex work), SW empowerment and other client-tailored interventions that take into account the economic pressures that SWs face and are bound to respond to. Future studies could employ a panel design or a difference in difference approach to adequately understand the causal effect of a major sporting event on the price of sex, quantity of sex acts supplied and the riskiness of the sex acts.

## Funding

This study acknowledges funding from 10.13039/100006661UNFPA and the 10.13039/100010269Wellcome Trust (#212347).

## CRediT authorship contribution statement

**Jacob Kazungu:** Conceptualization, Methodology, Formal analysis, Data curation, Writing – original draft, Writing – review & editing, Funding acquisition. **Marlise Richter:** Conceptualization, Investigation, Writing – review & editing, Funding acquisition. **Stanley Luchters:** Investigation, Writing – review & editing. **Matthew F. Chersich:** Conceptualization, Investigation, Writing – review & editing, Funding acquisition. **Matthew Quaife:** Conceptualization, Methodology, Formal analysis, Data curation, Writing – original draft, Writing – review & editing, Supervision.

## Declaration of competing interest

The authors declare that they have no known competing financial interests or personal relationships that could have appeared to influence the work reported in this paper.
